# Differential roles of Aβ42/40, p-tau231 and p-tau217 for Alzheimer’s trial selection and disease monitoring

**DOI:** 10.1038/s41591-022-02074-w

**Published:** 2022-12-01

**Authors:** Nicholas J. Ashton, Shorena Janelidze, Niklas Mattsson-Carlgren, Alexa Pichet Binette, Olof Strandberg, Wagner S. Brum, Thomas K. Karikari, Fernándo González-Ortiz, Guglielmo Di Molfetta, Francisco J. Meda, Erin M. Jonaitis, Rebecca Langhough Koscik, Karly Cody, Tobey J. Betthauser, Yan Li, Eugeen Vanmechelen, Sebastian Palmqvist, Erik Stomrud, Randall J. Bateman, Henrik Zetterberg, Sterling C. Johnson, Kaj Blennow, Oskar Hansson

**Affiliations:** 1 Department of Psychiatry and Neurochemistry, Institute of Neuroscience and Physiology, the Sahlgrenska Academy at the University of Gothenburg, Mölndal, Sweden; 2 King’s College London, Institute of Psychiatry, Psychology and Neuroscience, Maurice Wohl Institute Clinical Neuroscience Institute, London, UK; 3 NIHR Biomedical Research Centre for Mental Health and Biomedical Research Unit for Dementia at South London and Maudsley NHS Foundation, London, UK; 4 Centre for Age-Related Medicine, Stavanger University Hospital, Stavanger, Norway; 5 Clinical Memory Research Unit, Faculty of Medicine, Lund University, Lund, Sweden; 6 Department of Neurology, Skåne University Hospital, Lund University, Lund, Sweden; 7 Wallenberg Center for Molecular Medicine, Lund University, Lund, Sweden; 8 Graduate Program in Biological Sciences: Biochemistry, Universidade Federal do Rio Grande do Sul (UFRGS), Porto Alegre, Brazil; 9 Department of Psychiatry, University of Pittsburgh, Pittsburgh, PA USA; 10 Wisconsin Alzheimer’s Institute, School of Medicine and Public Health, University of Wisconsin, Madison, WI USA; 11 Wisconsin Alzheimer’s Disease Research Center, School of Medicine and Public Health, University of Wisconsin, Madison, WI USA; 12 Department of Neurology, Washington University School of Medicine, St. Louis, MO USA; 13 SILQ Center, Washington University School of Medicine, St. Louis, MO USA; 14 ADx NeuroSciences, Technologiepark 94, Ghent, Belgium; 15 Memory Clinic, Skåne University Hospital, Malmö, Sweden; 16 Clinical Neurochemistry Laboratory, Sahlgrenska University Hospital, Mölndal, Sweden; 17 Department of Neurodegenerative Disease, UCL Institute of Neurology, Queen Square, London, UK; 18 UK Dementia Research Institute at UCL, London, UK; 19 Hong Kong Center for Neurodegenerative Diseases, Hong Kong, China

**Keywords:** Diagnostic markers, Predictive markers

## Abstract

Blood biomarkers indicative of Alzheimer’s disease (AD) pathology are altered in both preclinical and symptomatic stages of the disease. Distinctive biomarkers may be optimal for the identification of AD pathology or monitoring of disease progression. Blood biomarkers that correlate with changes in cognition and atrophy during the course of the disease could be used in clinical trials to identify successful interventions and thereby accelerate the development of efficient therapies. When disease-modifying treatments become approved for use, efficient blood-based biomarkers might also inform on treatment implementation and management in clinical practice. In the BioFINDER-1 cohort, plasma phosphorylated (p)-tau231 and amyloid-β42/40 ratio were more changed at lower thresholds of amyloid pathology. Longitudinally, however, only p-tau217 demonstrated marked amyloid-dependent changes over 4–6 years in both preclinical and symptomatic stages of the disease, with no such changes observed in p-tau231, p-tau181, amyloid-β42/40, glial acidic fibrillary protein or neurofilament light. Only longitudinal increases of p-tau217 were also associated with clinical deterioration and brain atrophy in preclinical AD. The selective longitudinal increase of p-tau217 and its associations with cognitive decline and atrophy was confirmed in an independent cohort (Wisconsin Registry for Alzheimer’s Prevention). These findings support the differential association of plasma biomarkers with disease development and strongly highlight p-tau217 as a surrogate marker of disease progression in preclinical and prodromal AD, with impact for the development of new disease-modifying treatments.

## Abstract (web summary)

Longitudinal study of plasma biomarkers, in two independent cohorts, highlighted only p-tau217 as a surrogate marker of disease progression in early Alzheimer’s disease, supporting the development of new disease-modifying treatments.

## Main

The accumulation of amyloid-β (Aβ) peptides, sequestered into extracellular plaques, and intracellular neurofibrillary tangles comprising tau protein are the defining criteria of AD. These pathologies can be identified in vivo by cerebrospinal fluid (CSF) and positron emission tomography (PET) biomarkers^1^. Drug trials for AD are increasingly incorporating these biomarkers as necessary inclusion criterion and evidence of target engagement. However, in the early stages of AD, when individuals with notable cerebral Aβ accumulation are nonsymptomatic or present with subjective or mild cognitive complaints, trials are hindered by difficulties in determining drug effects on clinically relevant outcomes. Biomarkers that reflect key pathophysiological processes related to the drug target, or mechanisms putatively downstream of the drug target (for example, tau pathology or neurodegeneration for an anti-amyloid treatment) could be used to inform on promising disease-modifying therapies. Ideal biomarkers for enrichment or inclusion should have large effect sizes at baseline to identify suitable trial participants. In contrast, optimal biomarkers for longitudinal monitoring should have a large degree of change over time, which is specific to AD pathology and not observed in those without such pathology (for example, healthy elderly, or other neurodegenerative diseases). As highlighted in the recent Alzheimer’s Association Appropriate Use recommendation for use of AD biomarkers^2^, these changes in longitudinal measures of blood biomarkers should also be associated with established measures of AD progression, including worsening in objective cognitive performance and atrophy in brain regions known to be affected by the disease. In future clinical practice, when disease-modifying treatments are approved and are readily available, dynamic biomarkers that either track disease progression, or change towards normalization with efficient treatment, might potentially also be used to follow treatment effects and inform on decisions to initiate, suspend or restart treatment.

For both trial design purposes and future applications in clinical practice, it is beneficial if biomarkers are based on blood rather than CSF or PET, to increase availability and diversity, while reducing overall recruitment time and cost. Recently, blood biomarkers reflecting Aβ^3,4^, tau^5–8^, neurodegeneration^9,10^ and astrogliosis^11,12^, have been developed and validated. These markers, in particular different variants of phosphorylated tau (p-tau), exhibit high performance in identifying AD pathology in the differential diagnosis of cognitive decline and demonstrate excellent prognostic performance to predict progression to AD dementia^13^. In addition, p-tau variants in blood have been validated against neuropathology exhibited at postmortem^5,6,14–16^. Thus, blood biomarkers offer a noninvasive and widely available assessment to accurately identify AD at all disease stages. Now, to aid disease-modifying trials, studies are needed to establish the meaning of blood biomarker change in response to incipient AD pathology and identify plasma biomarkers that accurately reflect meaningful longitudinal brain atrophy and cognitive deterioration. Developing evidence suggests that changes of plasma Aβ42/40 (ref. ^3^) and p-tau (refs. ^5,17,18^^19–21^) are elevated in preclinical disease and might act as an integral enrichment aid for AD trials. In addition, plasma neurofilament light (NfL) and glial acidic fibrillary protein (GFAP) have been shown to be increased in preclinical (GFAP^12^) and prodromal (NfL^10^) stages of AD, respectively. Nevertheless, it is not known which of several recently developed high-performing blood biomarkers has the best performance for clinical trial selection and monitoring in future clinical practice.

Therefore, in this study, from two independent cohorts, we compared plasma biomarkers (p-tau181, p-tau217, p-tau231, Aβ42/40, GFAP and NfL) for the optimal identification of Aβ pathology in the early stages of AD (preclinical and mild cognitive impairment (MCI)). In addition, and importantly, we examined whether certain plasma biomarkers specifically change over time in those with confirmed Aβ pathology and assessed if these longitudinal changes also associated with longitudinal changes in cognition and brain atrophy in preclinical AD.

## Results

### Study cohorts

This study consisted of both cross-sectional (cohort 1) and longitudinal (cohort 2 and cohort 3) analyses. In the cross-sectional analysis, the goal was to quantify biomarker performance to identify Aβ pathology in cognitively unimpaired participants (CU, *n* = 388) and patients with MCI (*n* = 187) (Extended Data Table 1). The first longitudinal analysis was performed in cohort 2 (CU, *n* = 147; MCI, *n* = 95), which was a subcohort of the participants from cohort 1 with up to 6 years of longitudinal plasma measures (a median of three samples per participant over a median 4.3 years), magnetic resonance imaging (MRI) and cognitive assessments (Extended Data Table 2). All participants included in cohorts 1 and 2 were recruited from the prospective and longitudinal BioFINDER-1 study (www.biofinder.se) from 2009 to 2014 in southern Sweden. No significant differences between the demographic and clinical data between the participants included in the cohorts 1 and 2 were observed (Extended Data Table 3). Lastly, we validated the longitudinal results in 161 CU participants of the independent North American cohort Wisconsin Registry for Alzheimer’s Prevention (WRAP) (cohort 3, Extended Data Table 4).

### Plasma biomarkers to identify Aβ pathology

**Fig. 1:**
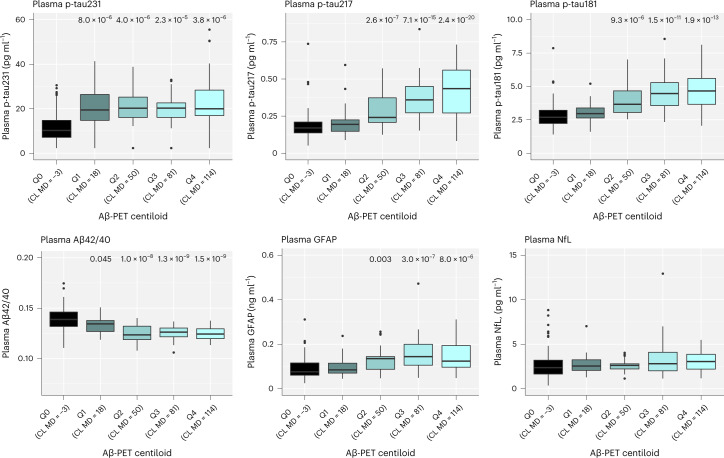
Associations between plasma biomarkers and Aβ-PET in BioFINDER-1 (cohort 1). Log10-transformed plasma biomarker levels were compared between the centiloid (CL) groups, <12 (MD −2.6; *n* = 139; reference group), Q1 (range 12.0–35.9; MD 17.9; *n* = 27), Q2 (range 35.9–71.7; MD 50.1; *n* = 24), Q3 (range 71.7–95.3; MD 80.6; *n* = 25) and Q4 (>95.3; MD 114.1; *n* = 25) using univariate general linear models adjusting for age. Untransformed data are presented in the boxplots to aid interpretation of biomarker values across different comparisons. One NfL outlier is not shown but was included in the statistical analysis. Boxes show interquartile range, the horizontal lines are medians and the whiskers were plotted using the Tukey method. Two-sided *P* values were corrected for multiple comparisons using Benjamini–Hochberg FDR; uncorrected and corrected *P* values are shown in Extended Data Table 6.

**Extended Data Fig. 1:**
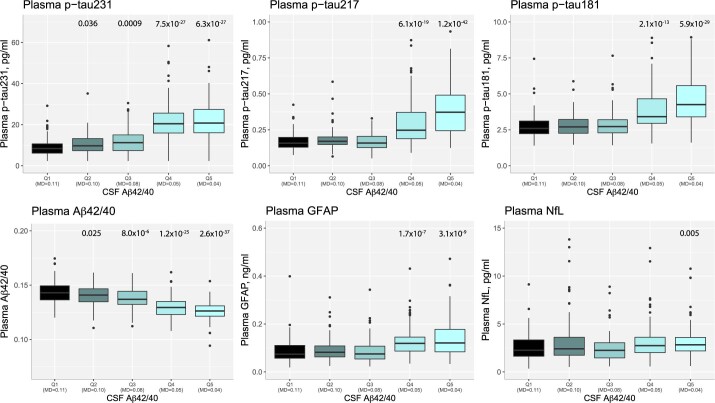
Associations between plasma biomarkers and CSF Aβ42/40 in BioFINDER-1 (cohort 1). Log10-transformed plasma biomarker levels were compared between the CSF Aβ42/40 quintile groups, Q1 ( > 0.102; median [MD], 0.108; n = 115, reference group), Q2 (range, 0.089-0.102; MD, 0.097; n = 115), Q3 (range 0.064-0.089; MD, 0.079; n = 115), Q4 (range, 0.042-0.064; MD, 0.051; n = 115) and Q5 (range, <0.042; MD, 0.035; n = 115) using univariate general linear models adjusting for age. Untransformed data are presented in the boxplots to aid interpretation of biomarker values across different comparisons. Outliers (p-tau217, n = 1; p-tau181, n = 2; GFAP, n = 1; NfL, n = 2) are not shown in the boxplots but were included in the statistical analysis. Boxes show interquartile range, the horizontal lines are medians, and the whiskers were plotted using Tukey method. Two-sided p-values were corrected for multiple comparisons using Benjamini–Hochberg false discovery rate; uncorrected and corrected p-values are shown in Extended Data Table 6.

In cohort 1, plasma p-tau231 had the highest area under the curve (AUC) to determine CU Aβ+ from CU Aβ− individuals (AUC = 0.854, 95% confidence interval (CI) 0.806 to 0.902) and had significantly higher accuracy than other plasma biomarkers, except for Aβ42/40 (AUC = 0.847, 95% CI 0.806 to 0.889) (Extended Data Table 5). In MCI patients, no significant differences between p-tau biomarkers to distinguish between Aβ+ from Aβ– individuals were observed (AUCs = 0.828–0.882) (Extended Data Table 5). Next, we analyzed plasma biomarkers when grouping participants by Aβ-PET centiloids, which is an established measure to increase comparability across Aβ-PET methods^22^. Here, we demonstrated that plasma p-tau231 and plasma Aβ42/40 significantly changed at lower threshold of PET centiloids (Fig. 1 and Extended Data Table 6) and CSF Aβ42/40 levels (Extended Data Fig. 1 and Extended Data Table 6) than other plasma biomarkers. Yet, in this cross-sectional investigation, both p-tau231 and Aβ42/40 reached a plateau and had no further changes in participants with more abnormal levels of Aβ pathology. In contrast, p-tau217 and p-tau181 demonstrated continued increases in participants with higher Aβ burden.

### Aβ-pathology-dependent longitudinal changes in plasma biomarkers

**Fig. 2:**
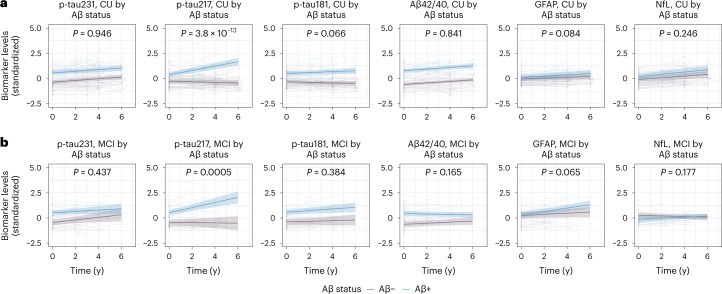
Longitudinal plasma biomarker changes in BioFINDER-1 (cohort 2). **a**,**b**, Longitudinal plasma biomarker changes stratified by β-amyloid status (negative, purple; positive, blue) in CU (**a**) and MCI (**b**). The *x* axis shows time from first plasma biomarker sample. Shaded areas represent 95% confidence intervals of the regression lines plotted from linear mixed effects models with the interaction between time and Aβ status as well as baseline Aβ status as independent variables and adjusting for age and sex. All p-tau biomarkers and Aβ42/40 were significantly changed in Aβ+ individuals at baseline in both CU and MCI (*P* < 0.001). Two-sided *P* values were corrected for multiple comparisons using Benjamini–Hochberg FDR; corrected and uncorrected *P* values are shown in Table 1 and Supplementary Table 1. Several outliers (p-tau231, *n* = 1; p-tau217, *n* = 9; p-tau181, *n* = 5; GFAP, *n* = 4; NfL, *n* = 4) are not shown but these data were included in the statistical analysis.

In cohort 2, we first tested for effects of baseline Aβ status on longitudinal plasma biomarker levels, for CU (Fig. 2a) and MCI participants (Fig. 2b), as summarized in Table 1 and Extended Data Table 7. Uncorrected *P* values for the results in Table 1 are presented in Supplementary Table 1. Only plasma p-tau217 had longitudinal increases over time in Aβ+ individuals in comparison with Aβ– individuals (time × Aβ-interaction: β = 0.249, *P* < 0.001). Likewise in MCI patients, only p-tau217 significantly increased in the Aβ+ group over time compared with the Aβ– group (time × Aβ-interaction: β = 0.270, *P* < 0.001).

**Table 1:** Associations of Aβ status with longitudinal plasma biomarker levels in BioFINDER-1 and WRAP

	BioFINDER-1 Cognitively unimpaired^a^	BioFINDER-1 Mild cognitive impairment^a^	WRAP Cognitively unimpaired^b^
Plasma biomarkers	Time × Aβ interaction β estimate (*P* value)		
p-tau231	−0.002 (0.946)	−0.063 (0.437)	−0.001 (0.951)
p-tau217	0.249 (3.8 × 10^−13^)	0.270 (0.0005)	0.103 (5.4 × 10^−8^)
p-tau181	0.073 (0.066)	0.050 (0.384)	0.047 (0.036)
Aβ42/40	0.007 (0.841)	−0.076 (0.165)	−0.019 (0.345)
GFAP	0.028 (0.084)	0.113 (0.065)	0.023 (0.273)
NfL	0.035 (0.246)	0.084 (0.177)	−0.039 (0.273)

β estimates and *P* values are from linear mixed effects models with the interaction between time and Aβ status as the independent variable, adjusted for age and sex. Two-sided *P* values were adjusted for multiple comparisons (*n* = 24, BioFINDER-1; *n* = 12, WRAP) using Benjamini–Hochberg FDR. Data tables with uncorrected *P* values are displayed in Supplementary Table 1.

^a^ Aβ42/40 data were available for 130 CU and 82 MCI; GFAP data were available for 124 CU and 82 MCI; NfL data were available for 125 CU and 82 MCI in BioFINDER-1. ^b^Data values for two participants were missing for p-tau231, p-tau181, Aβ42/40, GFAP and NfL in WRAP.

### Longitudinal changes in plasma biomarkers and longitudinal changes in cognition and atrophy

**Fig. 3:**
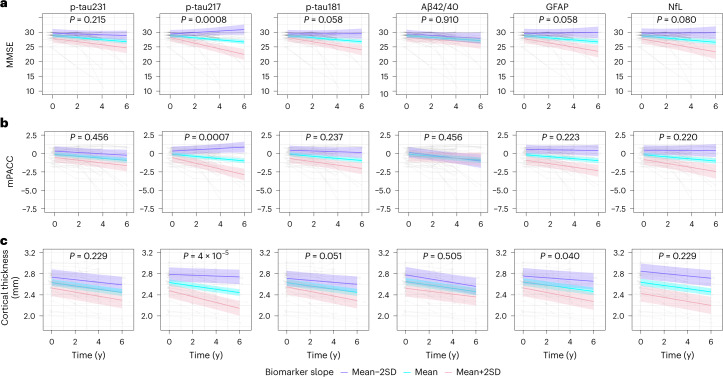
Associations of longitudinal plasma biomarkers with longitudinal cognitive decline and brain atrophy in BioFINDER-1 (cohort 2). **a**–**c**, The association between longitudinal plasma biomarkers and MMSE (**a**), mPACC (**b**) and cortical thickness of the typical AD signature regions (**c**) in Aβ positive cognitively unimpaired participants. The *x* axis shows time from first plasma biomarker samples. The model trajectories, shown as the mean slope and the mean ± 2 SD with 95% CI (shaded area), were plotted from linear mixed effects models with the interaction between time and standardized plasma biomarker slopes (derived from subject-level linear regression models) as an independent variable adjusting for age and sex; associations with cognition were also adjusted for years of education. Two-sided *P* values were corrected for multiple comparisons using Benjamini–Hochberg FDR; corrected and uncorrected *P* values are shown in Table 2 and Supplementary Table 2.

We further tested the associations between longitudinal changes of plasma biomarkers levels and longitudinal changes of global cognition and brain atrophy, indexed by Mini Mental State Examination (MMSE, Fig. 3a), Preclinical Alzheimer’s disease Cognitive Composite (mPACC, Fig. 3b) and cortical thickness of the typical AD signature regions (Fig. 3c), respectively, in Aβ + CU participants. Longitudinal change in plasma p-tau217 levels over time was significantly associated with worsening of MMSE (β = −0.308, *P* = 0.0008, Table 2), mPACC (β = −0.121, *P* = 0.0007, Table 2) and accelerated atrophy of cortical thickness over 6 years (β = −0.012, *P* < 0.001, Table 2). There was also a weak association between longitudinal GFAP and brain atrophy (β = −0.007, *P* = 0.040, Table 2). Uncorrected *P* values for the results in Table 2 are presented in Supplementary Table 2. When using both slopes of plasma p-tau217 and slopes of plasma GFAP simultaneously to predict longitudinal atrophy, plasma p-tau217 remained significant (*P* = 0.002), while the effect of plasma GFAP was attenuated (*P* = 0.77), suggesting that plasma GFAP did not contribute as a longitudinal proxy of atrophy beyond the effect of plasma p-tau217 in the early stages of AD. In addition to MMSE and mPACC, we also used a test of delayed recall memory, where only the slope of p-tau217 was significantly associated with cognitive decline (Extended Data Table 8). Results were very similar in a sensitivity analysis excluding samples below the lower limit of detection (Supplementary Tables 3–6).

**Table 2:** Associations between longitudinal plasma biomarkers and longitudinal MMSE, mPACC and cortical thickness of the typical AD signature regions in Aβ-positive cognitively unimpaired participants in BioFINDER-1 and WRAP

	BioFINDER-1			WRAP		
	MMSE^a^	mPACC^a^	Cortical thickness of the typical AD signature regions^b^	MMSE ^c^	mPACC ^c^	Cortical thickness of the typical AD signature regions ^d^
Plasma biomarkers	β estimate (*P* value)					
p-tau231	−0.100 (0.215)	−0.022 (0.456)	−0.004 (0.229)	−0.030 (0.737)	−0.020 (0.427)	−0.002 (0.363)
p-tau217	−0.308 (0.0008)	−0.121 (0.0007)	−0.012 (4.1 × 10^−5^)	−0.135 (0.003)	−0.098 (9.0 × 10^−7^)	−0.005 (0.021)
p-tau181	−0.180 (0.058)	−0.044 (0.237)	−0.006 (0.051)	−0.075 (0.215)	−0.030 (0.317)	0.004 (0.148)
Aβ42/40	−0.010 (0.910)	0.032 (0.456)	0.002 (0.505)	−0.014 (0.754)	−0.032 (0.317)	−0.003 (0.245)
GFAP	−0.198 (0.058)	−0.054 (0.223)	−0.007 (0.040)	0.013 (0.754)	−0.007 (0.745)	−0.004 (0.051)
NfL	−0.194 (0.080)	−0.067 (0.220)	−0.004 (0.229)	−0.046 (0.527)	−0.024 (0.379)	−0.004 (0.034)

β estimates and *P* values are from linear mixed effects models with the interaction between time and standardized plasma biomarker slopes (derived from subject-level linear regression models) as the independent variable, adjusted for age and sex; associations with cognition were also adjusted for years of education. Two-sided *P* values were adjusted for multiple comparisons within each variable (*n* = 6) using Benjamini–Hochberg FDR. Data tables with uncorrected *P* values are displayed in Supplementary Table 2.

^a^ Longitudinal MMSE, mPACC and plasma biomarker data were available for 57 (p-tau) and 49 (Aβ42/40, GFAP and NfL) Aβ-positive cognitively unimpaired BioFINDER-1 participants. ^b^ Longitudinal cortical thickness of the typical AD signature regions and plasma biomarker data were available for 56 (p-tau) and 48 (Aβ42/40, GFAP and NfL) Aβ-positive cognitively unimpaired BioFINDER-1 participants. ^c^ Longitudinal MMSE, mPACC and plasma biomarker data were available for 66 (p-tau217) and 65 (other biomarkers) Aβ-positive cognitively unimpaired participants in WRAP. ^d^Longitudinal cortical thickness of the typical AD signature regions and plasma biomarker data were available for 65 Aβ-positive cognitively unimpaired participants in WRAP.

### Validation of longitudinal analyses

**Fig. 4:**
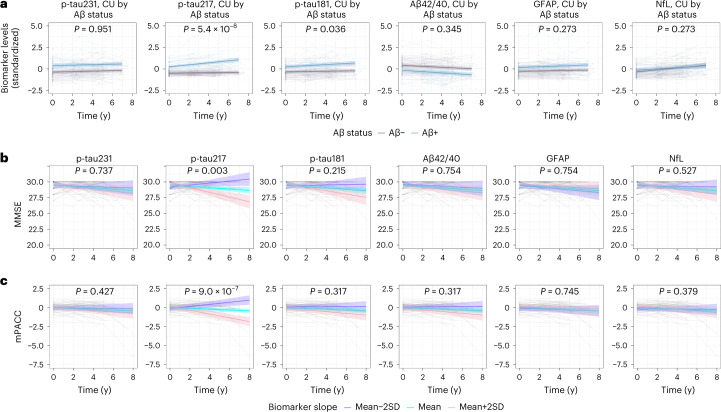
Longitudinal plasma biomarker changes and their association with cognitive decline in WRAP (cohort 3). **a**, Longitudinal plasma biomarker change stratified by Aβ status (negative, purple; positive, blue) in CU participants. The average regression lines with 95% CI (shaded area) were plotted from linear mixed effects models with the interaction between time and Aβ status as well as baseline Aβ status independent variables and adjusting for age and sex. All p-tau biomarkers, Aβ42/40 (*P* < 0.001) and GFAP (*P* = 0.005) were significantly changed in Aβ+ individuals at baseline. Several outliers (p-tau217, *n* = 5; p-tau181, *n* = 2; GFAP, *n* = 1; NfL, *n* = 3) are not shown in (**a**) but these data were included in the statistical analysis. **b**,**c**, The association between longitudinal plasma biomarkers and MMSE (**b**) and mPACC (**c**) in Aβ-positive cognitively unimpaired participants. The model trajectories, shown as the mean slope and the mean ± 2 SD with 95% CI (shaded area), were plotted from linear mixed effects models with the interaction between time and standardized plasma biomarker slopes (derived from subject-level linear regression models) as an independent variable adjusting for age, sex and years of education. Two-sided *P* values were corrected for multiple comparisons using Benjamini–Hochberg FDR; corrected and uncorrected *P* values are shown in Tables 1 and 2 and Supplementary Tables 1 and 2. One outlier with MMSE value of 17 is not shown in **b** but was included in the statistical analysis The *x* axes in **a**–**c** show time from first plasma biomarker samples.

**Extended Data Fig. 2:**
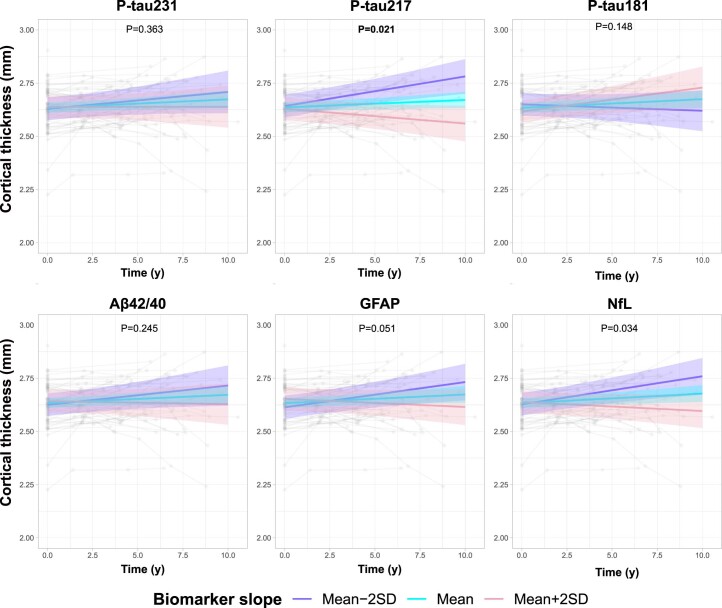
Associations between longitudinal plasma biomarkers and brain atrophy in WRAP (cohort 3). The association between longitudinal plasma biomarkers and cortical thickness of the typical AD signature regions in Aβ positive cognitively unimpaired participants. The x-axis show time from first plasma biomarker samples. The model trajectories, shown as the mean slope and the mean±2 SD with 95% CI (shaded area), were plotted from linear mixed effects models with the interaction between time and standardized plasma biomarker slopes (derived from subject-level linear regression models) as the independent variable adjusting for age and sex. Two-sided p-values were corrected for multiple comparisons using Benjamini–Hochberg false discovery rate; corrected and uncorrected p-values are shown in Table 2 and Supplementary Table 2.

Finally, we validated the longitudinal BioFINDER-1 findings in 161 CU participants from the WRAP cohort (cohort 3). Again, only p-tau217 increased substantially in Aβ+ individuals in comparison with Aβ– participants over 8 years (β = 0.103, *P* ≤ 0.001, Fig. 4a and Table 1). In contrast to the longitudinal BioFINDER-1 results, plasma p-tau181 also showed a significant, but modest, increase in Aβ+ individuals (β = 0.047, *P* = 0.036). Within Aβ + CU individuals, however, only the longitudinal increase in plasma p-tau217, but no observed changes of other plasma biomarkers, was significantly associated with declining cognition, as measured with longitudinal MMSE (β = −0.135, *P* = 0.003, Fig. 4b and Table 2), mPACC (β = −0.098, *P* < 0.001, Fig. 4c and Table 2) and a test of delayed recall memory over 8 years (β = −0.298, *P* < 0.001, Extended Data Table 8). Longitudinal changes in cortical thickness of typical AD signature regions were associated with longitudinal p-tau217, GFAP and NfL (Extended Data Fig. 2 and Table 2). However, when using slopes of these three biomarkers simultaneously to predict longitudinal atrophy, plasma p-tau217 remained significant (*P* = 0.016), while the effects of plasma GFAP and NfL were attenuated (*P* = 0.91 and *P* = 0.06, respectively).

## Discussion

The main finding of this study, which compared several state-of-the-art plasma biomarkers in early stages of AD, was that the longitudinal trajectory of plasma p-tau217, but not other candidate biomarkers, was closely related to disease progression. The significant and dynamic longitudinal changes in plasma p-tau217 correlated with changes in multiple domains of cognition and cortical thickness of typical AD signature regions. Specific other biomarkers (p-tau231 and Aβ42/40) had somewhat more pronounced cross-sectional changes in response to early Aβ pathology but did not change in the longitudinal analysis. Taken together, our results add to previous studies which have shown that plasma biomarkers can identify AD pathology, predict future dementia risk, and are associated with in vivo amyloid and tau pathologies^1^. The longitudinal changes in plasma p-tau217 suggest that this biomarker should be evaluated in interventional studies as an indicator of therapeutic effects in early stages of AD, as successful disease modification may be expected to be associated with reversion towards normal values for plasma p-tau217, rather than a continuing increase seen in untreated patients.

Our findings support the view that there are important differences in how plasma biomarkers represent AD-related processes. For example, while all p-tau biomarkers relate to AD postmortem pathology^6,14,15^, in AD brain tissue, p-tau217 is prominently seen in granulovacuolar degeneration bodies and multivesicular bodies in neurons, which is not observed for p-tau181 and p-tau231 (ref. ^23^). Such differences in neuropathological properties may be related to the different trajectories of different plasma biomarkers. Our results also support the developing evidence that among the most promising plasma AD biomarkers, p-tau231 and Aβ42/40 might have the earliest changes at the incipient stages of Aβ accumulation^5,17,18^. However, p-tau217 is also changing notably in preclinical AD^18,20^. Interestingly, p-tau231, and Aβ42/40 are not more changed in individuals with more advanced Aβ pathology, and a plateau is observed, particularly for p-tau231, at a phase when p-tau217 is continuing to increase. This cross-sectional observation is corroborated by our new longitudinal data, from two independent cohorts, demonstrating that longitudinal increases of p-tau217 in Aβ+ individuals are associated with worsening cognitive performance and brain atrophy in preclinical AD. Such independent associations were not observed for any other plasma biomarker tested in this study. This includes a marker of global neurodegeneration, plasma NfL, which has been shown to associate with clinical progression in patients with more advanced symptoms^24,25^. However, in our longitudinal preclinical data, this association between plasma NfL and disease progression is not observed. Our results confirm that p-tau217 is dynamic biomarker, even in preclinical AD^19^, accurately reflecting the progression of AD pathology, and now this is shown in comparison with a compendium of blood biomarkers also reported to reflect AD pathophysiology. The early changes of all p-tau plasma biomarkers, suggest that they are initially reflective of Aβ dysmetabolism^26^. However, over time, p-tau217 is the only biomarker that clearly changes with disease progression, which is in line with earlier observations that p-tau217 may later become more reflective of tau pathology, after the initial deposition of Aβ^27^. In symptomatic AD, several studies find similar diagnostic accuracy of p-tau181 and p-tau217 (refs. ^28,29^); however, most reports demonstrate larger fold-changes for p-tau217 (ref. ^28^). This is likely attributed to the longitudinal and dynamic increase of p-tau217 shown in this study, which is associated with metrics of AD progression. Data from CSF studies have also shown that p-tau217 exhibits larger fold-changes in symptomatic phases^30^, while subtle changes of p-tau231 are observed with regional Aβ deposition^31^ and these results now translate to blood.

Our results on baseline performance for biomarkers to detect Aβ pathology are promising for the use of plasma biomarkers as instruments to guide selection of participants into clinical trials. The results for longitudinal changes in plasma p-tau217 provide rationale for future analyses in clinical trials to determine whether treatment-induced reductions in plasma p-tau217 towards normal values are clearly associated with clinical beneficial effects. If such a relationship can be established in clinical trials, future trials targeting early-stage AD might incorporate plasma p-tau217 as a potential surrogate endpoint^2^. Importantly, a recent clinical trial evaluating donanemab, an immunotherapy efficiently removing Aβ aggregates from the brain, has shown 23% reduction in levels of plasma p-tau217 within 6–18 months of treatment when the placebo group continued to increase^32^.

In a longer perspective, our results may also be important for clinical practice. It is possible that one or several disease-modifying treatments against AD will become widely available for clinical use within a few years. This will bring an urgent need to make informed clinical decisions in millions of patients. Biomarkers will then be required to both identify AD and track progression of the disease with objective measures. This need will quickly overcome the available PET and CSF resources in healthcare systems worldwide, and blood biomarkers will be essential. Future clinical studies that include active interventions are warranted to best determine how to incorporate longitudinal blood biomarker measures into clinical workflows, for example, studying whether a disease-modifying treatment could be temporarily halted when plasma p-tau217 vales have been normalized. Further, the longitudinal results of the current study suggest that plasma p-tau217 is a key biomarker to be used when assessing already banked samples from performed clinical trials, which have evaluated relevant therapies or lifestyle interventions, to determine whether such treatments affect the development of AD-related pathology.

Although this study is the largest that simultaneously tests several state-of-the-art and relevant plasma biomarkers for AD in early disease stages with a longitudinal design, the sample sizes in the longitudinal analyses of BioFINDER-1 were still relatively small. Therefore, it was essential that such longitudinal findings were independently replicated in the WRAP cohort. Still, larger studies on more heterogenous populations are needed to confirm the relative differences in biomarker trajectories before firm conclusions can be drawn for the preferential use of longitudinal measures of certain plasma biomarkers in clinical practice and trials. We acknowledge that the assay designs (for example, antibody and/or analytical platform differences) also have different performances and may have contributed to our findings. For example, p-tau231 and p-tau217 assays have different properties of sensitivity due to healthy individuals being below the lower limit of detection more often for the p-tau217 measurements^33^. Other p-tau217 assays may have more sensitive performance at the earliest changes of Aβ-PET^34^. Lastly, plasma biomarker studies published to date are heavily weighted towards Caucasian participants. A recent pilot report demonstrated that plasma p-tau231 and p-tau181 were less accurate for detecting abnormalities in Aβ pathology in an African American population^35^. Yet, p-tau217 has shown good diagnostic accuracy in diverse multiethnic populations^36^. Therefore, establishing whether the longitudinal trajectories and response to early Aβ dysmetabolism of blood biomarkers can be directly translated to different populations warrants detailed investigation.

In conclusion, plasma AD biomarkers may offer complementary information as noninvasive, widely accessible and impartial measures for improved design of clinical trials. Incorporation of these measures in clinical trial design may accelerate the development and implementation of successful prevention and treatment of AD. Plasma p-tau231, Aβ42/40 and p-tau217 appear to be biomarkers changing early in response to Aβ pathology. Our cross-sectional data suggest earlier changes for p-tau231 and Aβ42/40 which should be explored further as screening tools for preclinical Aβ deposition. However, in terms of monitoring dynamic disease progression, plasma p-tau217 has clear advantages due to its continued increase during the early disease development and associations to AD measures of cognitive decline and brain atrophy which was not robustly observed for any other plasma biomarker. This supports the potential use of plasma p-tau217 as a surrogate outcome marker in ongoing and future intervention trials as well as for tracking disease progression in clinical practice.

## Methods

### Participants

All participants for cohort 1 and cohort 2 were recruited in the prospective and longitudinal BioFINDER-1 study (www.biofinder.se) from 2009 to 2014 in southern Sweden. The participants included CU participants (recruited as cognitively normal controls or as subjective cognitive decline patients) and patients with MCI. Details on recruitment, exclusion and inclusion criteria have been presented before^13,37,38^. All participants underwent lumbar puncture at baseline for CSF sampling. Plasma samples were taken at baseline and every second year for up to 6 years. Cognitive function was assessed with MMSE, Word list memory delayed recall (from the Alzheimer’s Disease Assessment Scale (ADAS-cog)) and mPACC. The mPACC was calculated as the average of five *z* scores for tests of global cognition (MMSE), memory (the word list delayed recall test from the cognitive subscale from the ADAS-cog, counted twice to preserve the weight on memory from the original PACC), executive function (Trail Making Test B) and verbal ability (animal fluency)^39–41^. All participants in cohort 3 were from WRAP. Design and assessments including cognitive battery of the WRAP study are described in detail elsewhere^42,43^. In brief, all participants were cognitively normal at first blood collection, recruited from the populations and enriched for positive parental history of AD and were between 40 and 65 years at baseline. The components of the mPACC were MMSE, the Logical Memory Delayed Recall test, the Trail Making Test B and the Rey Auditory Verbal Learning Test total score over five learning trials. The study was approved by the Regional Ethics Committee in Lund, Sweden. The WRAP data were collected under a University of Wisconsin-Madison Institutional Review Board protocol. All participants in all three cohorts gave their informed consent to participate in the study and the data were collected according to the Declaration of Helsinki.

### Biochemical analyses

CSF concentrations of Aβ42 and Aβ40 were determined using ELISA kits (Euroimmun) or the NeuroToolKit on Cobas e601 (Roche Diagnostics) in the BioFINDER-1 longitudinal and cross-sectional samples, respectively. CSF Aβ42/40 Euroimmun data were binarized using a threshold of 0.091 (ref. ^20^) and for NeuroToolKit CSF Aβ42/40 we used a threshold of 0.066 determined using mixture modeling. Plasma concentrations of p-tau217 and p-tau181 were measured using an immunoassay developed by Lilly Research Laboratories at Lund University^19,20^. Plasma p-tau231 was analyzed using in-house single molecular arrays (Simoa) developed at the University of Gothenburg^5^. Plasma concentrations of Aβ42 and Aβ40 were quantified using an immunoprecipitation-coupled mass spectrometry method developed at Washington University^4^. Plasma GFAP and NfL were analyzed using in-house Elecsys prototype plasma immunoassays (not commercially available) on Cobas e601 analyzers (Roche Diagnostics). Plasma concentrations of p-tau231, p-tau217 and p-tau181 were below the detection limit of the assay in 4.0%, 16.0% and 9.8% of the samples, respectively, which is in the same range as in previous studies^6,8^. In WRAP, p-tau217 and p-tau231 were analyzed using the same biochemical methods as the BioFINDER-1 cohort. Plasma p-tau181, Aβ42, Aβ40, GFAP and NfL were measured using a commercially available immunoassay from Quanterix (p-Tau-181 V2 Advantage Kit and Neurology 4-Plex E). In WRAP, plasma concentrations of p-tau231, p-tau217 and p-tau181 were below the detection limit of the assay in 0.2%, 1.2% and 0.2% of the samples, respectively.

### Neuroimaging

In BioFINDER-1, a 3T MRI scanner (Siemens Tim Trio 3T) was used for anatomical T1-weighted imaging. Magnetization-prepared rapid gradient-echo (MP-RAGE) images (repetition time (TR) = 1.950 ms, time to echo (TE) = 3.4 ms, 1 mm isotropic voxels, 178 slices) and the FreeSurfer image analysis pipeline v.6.0 (see http://surfer.nmr.mgh.harvard.edu/) were used in the anatomical segmentation and cortical thickness calculations^19^. For these analyses, we calculated cortical thickness (adjusted for surface area) from a temporal meta-region of interest, consisting of bilateral entorhinal, fusiform, inferior temporal and middle temporal cortex, which constitute the typical AD signature regions^44^. Aβ imaging was performed at baseline visit using [^18^F]flutemetamol PET^6^. Standardized uptake value ratio images were created using dynamic (list-mode) 90–100-min postinjection data and the whole cerebellum as reference region. Centiloids were derived using the Computational Analysis of PET from AIBL pipeline^45^. In WRAP, participants underwent T1-weighted MRI and amyloid [^11^C]-Pittsburgh Compound B (PiB) imaging^46–48^. Cortical thickness in the typical AD signature regions was determined using the same approach as in the BioFINDER-1 cohort. We included MRI scans performed within 2 years of any blood collection visit. Aβ burden was assessed as a global cortical average [^11^C]-PiB distribution volume ratios (DVR) and a threshold of DVR > 1.19 across eight bilateral regions of interest was used to define PiB positivity^48^.

### Statistical analyses

Baseline levels and longitudinal changes in standardized plasma biomarkers (*z* scores) were tested in linear mixed effects models with the interaction between time and Aβ status as well as baseline Aβ status as independent variables adjusted for age and sex (using the R *lme4* package). All biomarkers were standardized based on the corresponding mean and SD within analyzed groups. To study associations of longitudinal changes in plasma biomarkers with longitudinal cognition (for example, with MMSE, mPACC and Word list memory delayed recall (from ADAS-cog in BioFINDER-1 and Rey Auditory Verbal Learning Test in WRAP)) and cortical thickness of the typical AD signature regions, we used linear mixed effects models with the interaction between time and standardized plasma biomarker slopes (derived from subject-level linear regression models, with time as predictor of biomarker levels) as the independent variable, adjusted for age and sex. For cognition we also included years of education as a covariate. To facilitate biomarker comparisons, we used the inverse ratio of Aβ42 and Aβ40 in the longitudinal analysis. In cohort 1, study participants were classified as amyloid-negative using centiloid threshold of 12 (median (MD) –2.6; *n* = 139; reference group), which was chosen based on previous comparisons to both CSF Aβ42 and neuropathology^49–51^. Centiloids <12 are regarded as normal and represent signal noise. Individuals with centiloids >12 were further classified into the centiloid quartile groups Q1 (range 12.0–35.9; MD 17.9; *n* = 27), Q2 (range 35.9–71.7; MD 50.1; *n* = 24), Q3 (range 71.7–95.3; MD 80.6; *n* = 25) and Q4 (>95.3; MD 114.1; *n* = 25). In addition, participants in cohort 1 were classified into CSF Aβ42/40 quintile groups, Q1 (>0.102; MD 0.108; *n* = 115, reference group), Q2 (range 0.089–0.102; MD 0.097; *n* = 115), Q3 (range 0.064–0.089; MD 0.079; *n* = 115), Q4 (range 0.042–0.064; MD 0.051; *n* = 115) and Q5 (range <0.042; MD 0.035; *n* = 115). Plasma biomarker levels (log10-transformed) were compared between the centiloid groups (<12, Q1, Q2, Q3 and Q4) and CSF Aβ42/40 quintile groups (Q1, Q2, Q3, Q4 and Q5) using univariate general linear models adjusting for age (with centiloid <12 and CSF Aβ42/40 Q1 as reference groups). AUC of two ROC curves were compared with the DeLong test. *P* values adjusted for multiple comparisons using false discovery rate (FDR) were considered significant at *P* < 0.05, two-tailed. FDR correction was applied separately for each outcome measure with the numbers of comparisons shown in table footnotes. Statistical analyses were done in R (v.4.0.2) and SPSS (v.28).

### Reporting summary

Further information on research design is available in the Nature Portfolio Reporting Summary linked to this article.

## Online content

Any methods, additional references, Nature Portfolio reporting summaries, source data, extended data, supplementary information, acknowledgements, peer review information; details of author contributions and competing interests; and statements of data and code availability are available at 10.1038/s41591-022-02074-w.

### Supplementary information

Supplementary Information Supplementary Tables 1–6.

Reporting Summary

## Figures and Tables

**Extended Data Table 1:**
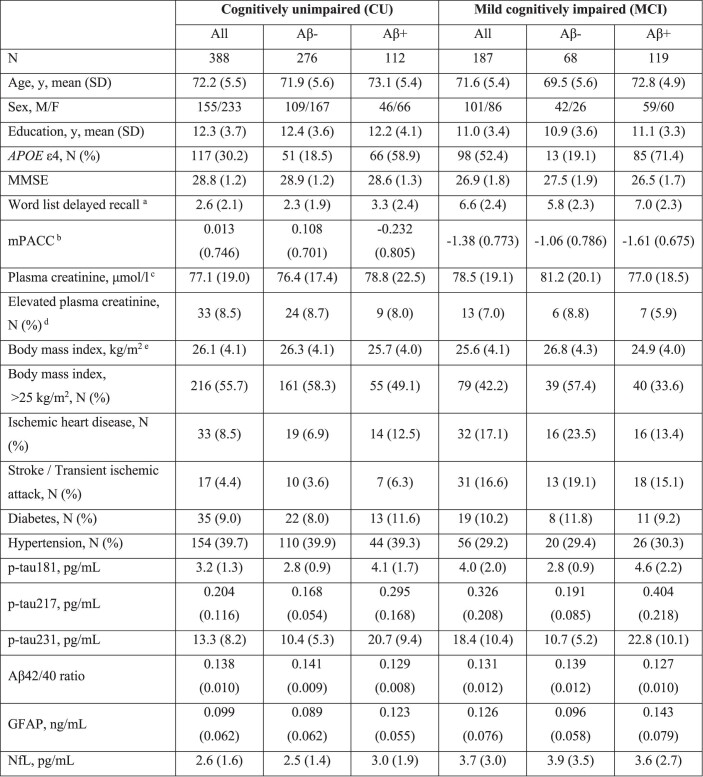
Demographics of cohort 1 (BioFINDER-1 cross-sectional sub-cohort)

Higher scores of MMSE and mPACC, but lower scores for word list delayed recall, mean better performance. ^a^ Word list memory delayed recall (from ADAS-cog) scores were missing for 2 CU and 11 MCI participants. ^b^ mPACC scores were missing for 39 CU and 45 MCI participants. ^c^Creatinine data were missing for 9 CU and 9 MCI MCI participants. ^d^ Creatinine levels above 90 μmol/l in women and above 105 μmol/l were considered as high. ^e^Body mass index data were missing for 15 CU and 26 MCI participants.

**Extended Data Table 2:**
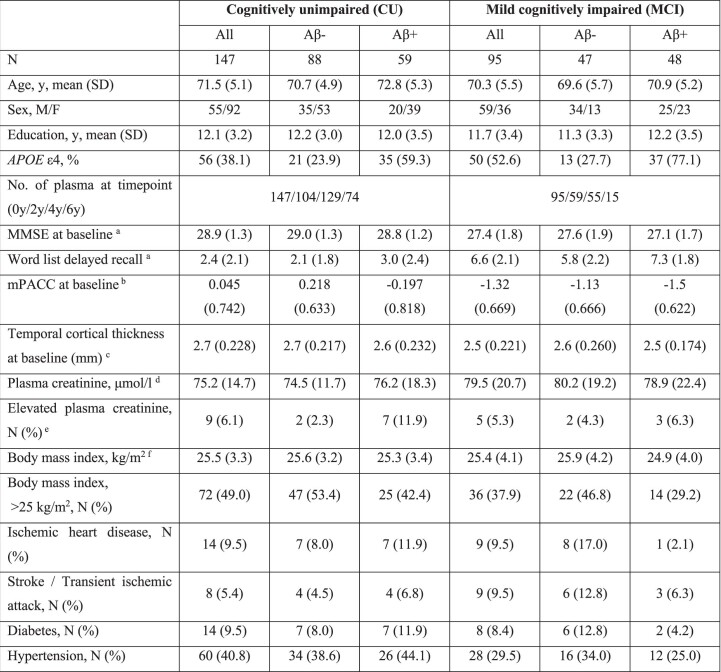
Demographics of cohort 2 (BioFINDER-1 longitudinal sub-cohort)

Higher scores of MMSE and mPACC, but lower scores for word list delayed recall, mean better performance. ^a^ Baseline MMSE and ADAS-cog scores were missing for 1 CU and 2 MCI participants. ^b^ Baseline mPACC scores were missing for 17 CU and 16 MCI participants. ^c^ Baseline cortical thickness data were missing for 19 CU and 9 MCI participants. ^d^Creatinine data were missing for 3 CU and 9 MCI MCI participants. ^e^ Creatinine levels above 90 μmol/l in women and above 105 μmol/l were considered as high. ^f^Body mass index data were missing for 2 CU and 13 MCI participants.

**Extended Data Table 3:**
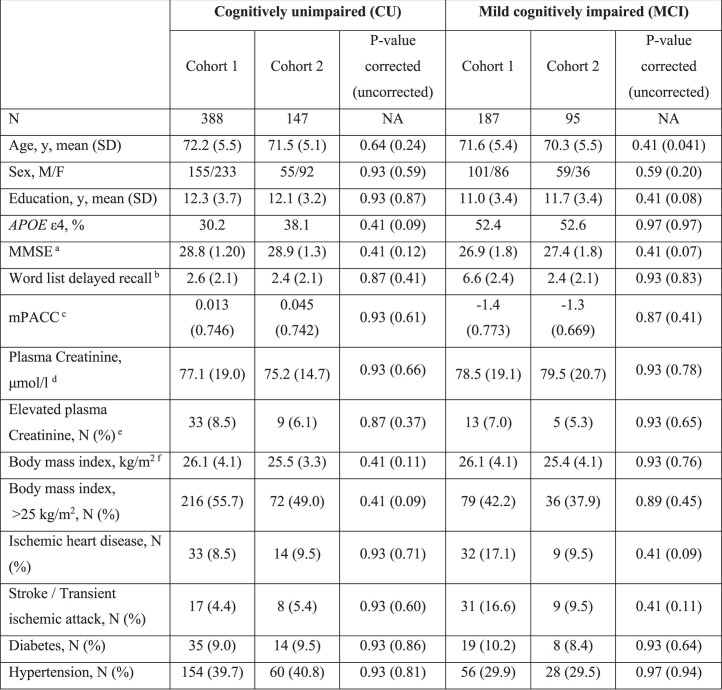
Comparison of cohort 1 (BioFINDER-1 cross-sectional sub-cohort) and cohort 2 (BioFINDER-1 longitudinal sub-cohort)

Two-sided p-values (when appropriate) are from Mann-Whitney or Chi-Square (sex, APOE ε4 status) tests. P-values were corrected for multiple comparisons (n = 30) using Benjamini–Hochberg false discovery rate. Higher scores of MMSE and mPACC, but lower scores for word list delayed recall, mean better performance. ^a^ MMSE scores were missing for 3 participants from cohort 2. ^b^ Word list delayed recall (from ADAS-cog) scores were missing for 13 participants from cohort 1 and 3 participants from cohort 2. ^c^ mPACC scores were missing for 84 participants from cohort 1 and 33 participants from cohort 2. ^d^Creatinine data were missing for 18 participants from cohort 1 and 12 participants from cohort 2. ^e^ Creatinine levels above 90 μmol/l in women and above 105 μmol/l were considered as high. ^f^Body mass index data were missing for 41 participants from cohort 1 and 15 participants from cohort 2.

**Extended Data Table 4:**
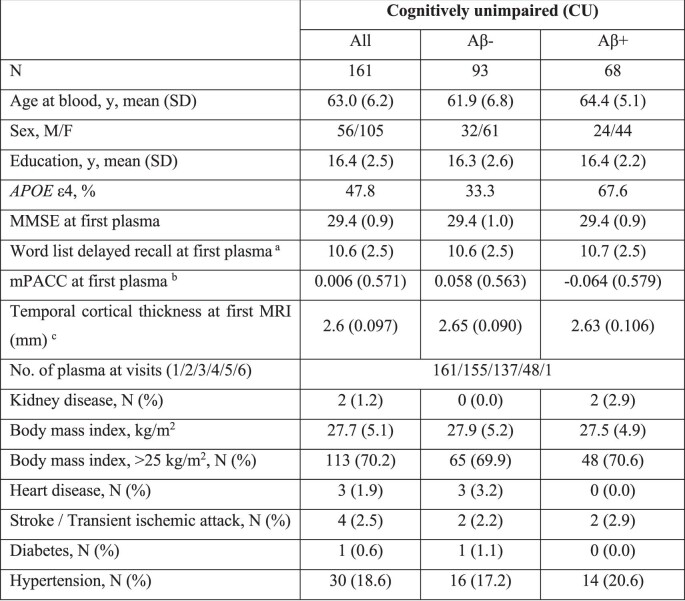
Demographics of cohort 3 (WRAP longitudinal cohort)

**Extended Data Table 5:**
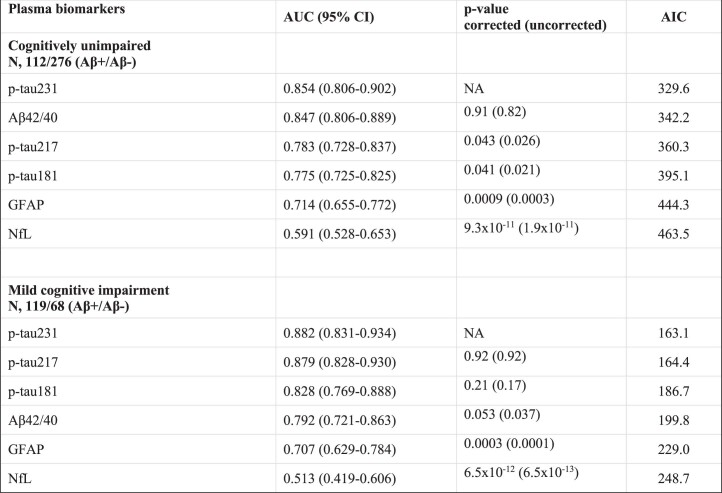
Discrimination of Aβ positive vs negative participants in BioFINDER-1 (cohort 1)

AUC of two ROC curves were compared with DeLong test. Two-sided p-values were adjusted for multiple comparisons (n = 10) using Benjamini–Hochberg false discovery rate. Aβ status was defined based on CSF Aβ42/40 binarized using a threshold of 0.066 determined using mixture modelling.

**Extended Data Table 6:**
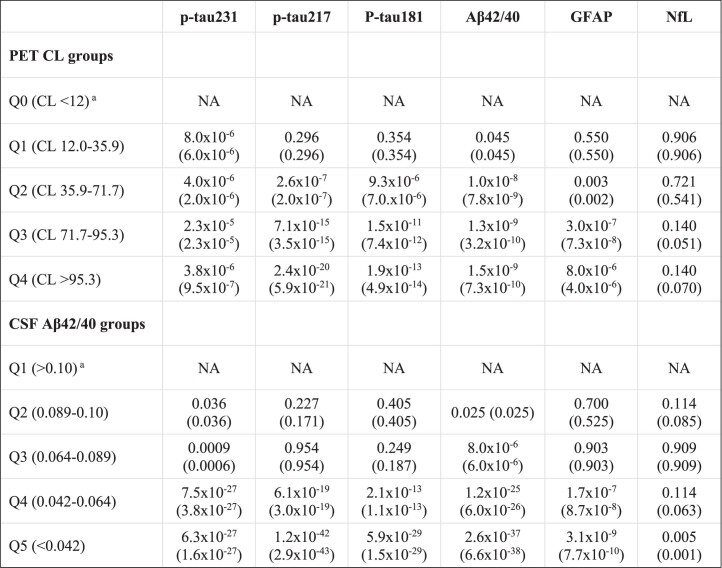
P-values for biomarkers comparisons in Fig. 1 and Extended Data Fig. 1

Data are corrected (uncorrected) two-sided p-values; p-values were corrected for multiple comparisons (n = 4) using Benjamini–Hochberg false discovery rate. ^a^ Reference group.

**Extended Data Table 7:**
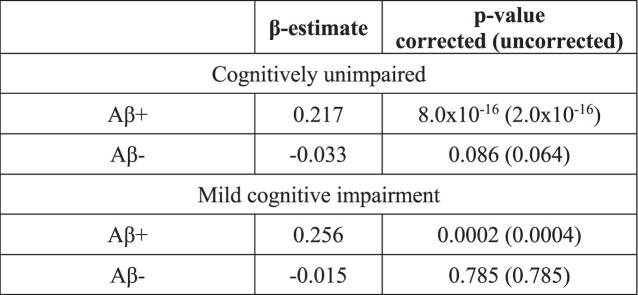
Longitudinal changes in plasma p-tau217 concentration over time by Aβ status in BioFINDER-1 (cohort 2)

β-estimates and two-sided p-values are from linear mixed effects models with time as the independent variable, adjusted for age and sex. P-values were adjusted for multiple (n = 4) comparisons using Benjamini–Hochberg false discovery rate.

**Extended Data Table 8:**
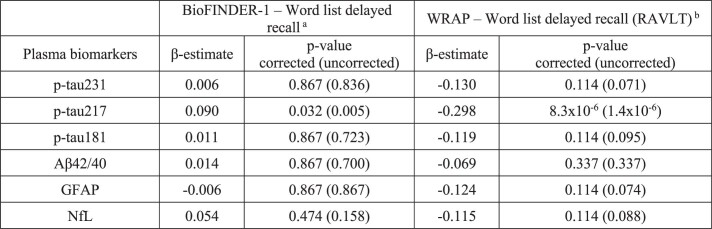
Associations between longitudinal plasma biomarkers and longitudinal Word list delayed recall and Rey Auditory Verbal Learning Test in Aβ positive cognitively unimpaired participants in BioFINDER-1 (cohort 2) and WRAP (cohort 3), respectively

β-estimates and two-sided p-values are from linear mixed effects models with the interaction between time and standardized plasma biomarker slopes (derived from subject-level linear regression models) as the independent variable, adjusted for age, sex and years of education. P-values were adjusted for multiple comparisons (n = 6) using Benjamini–Hochberg false discovery rate. ^a^ Longitudinal Word list delayed recall and plasma biomarker data were available for 57 (p-tau) and 49 (Aβ42/40, GFAP and NfL) Aβ positive cognitively unimpaired participants in BioFINDER-1. ^b^ Longitudinal RAVLT and plasma biomarker data were available for 66 (p-tau217) and 65 (other biomarkers) Aβ positive cognitively unimpaired participants in WRAP.

## Data Availability

Anonymized aggregated level data will be shared by request from a qualified academic investigator for the sole purpose of replicating procedures and results presented in the article, and as long as data transfer is in agreement with EU legislation on the general data protection regulation and decisions by the Ethical Review Board of Sweden and Region Skåne, which should be regulated in a material transfer agreement.
