# Supplementary material for: Differential roles of Aβ42/40, p-tau231 and p-tau217 for Alzheimer’s trial selection and disease monitoring
Source: Nat Med. 2022 Dec 1;28(12):2555–62. doi: 10.1038/s41591-022-02074-w (PMC9800279; doi:10.1038/s41591-022-02074-w)
Supplement: Supplementary file 1 — Supplementary Tables 1–6. [file 41591_2022_2074_MOESM1_ESM.pdf]

nature medicine

Article

https://doi.org/10.1038/s41591-022-02074-w

Differential roles of Aβ42/40, p-tau231 and p-tau217 for Alzheimer's trial selection and disease monitoring

In the format provided by the authors and unedited

Online Supplementary

Table of Contents – Online Supplementary

Supplementary Table 1 – Uncorrected p-values from Table 1 (Associations of Aβ status with longitudinal plasma biomarker levels in BioFINDER-1 and WRAP), **pg 2**

Supplementary Table 2 – Uncorrected p-values from Table 2(Associations between longitudinal plasma biomarkers and longitudinal mini mental state examination (MMSE), modified Preclinical Alzheimer's Cognitive Composite (mPACC), and cortical thickness of the typical AD signature regions in Aβ positive cognitively unimpaired participants in BioFINDER-1 and WRAP), **pg 2**

Supplementary Table 3 – Associations of plasma biomarkers with CSF Aβ42/40 (excluding plasma p-tau values below the detection limit) in BioFINDER-1 (cohort 1), **pg 3**

Supplementary Table 4 – P-values for biomarkers comparisons in Figure 1 excluding plasma p-tau values below the detection limit), **pg 3**

Supplementary Table 5 – Associations of Aβ status with longitudinal plasma biomarker levels (excluding plasma p-tau values below the detection limit) in BioFINDER-1 (cohort 2), **pg 4**

Supplementary Table 6 – Associations between longitudinal plasma biomarkers and longitudinal mini mental state examination (MMSE), modified Preclinical Alzheimer's Cognitive Composite (mPACC) and cortical thickness of the typical AD signature regions in Aβ positive cognitively unimpaired participants (excluding plasma p-tau values below the detection limit) in BioFINDER-1 (cohort 2), **pg 5**

Supplementary Table 1. Uncorrected p-values from Table 1 (Associations of Aβ status with longitudinal plasma biomarker levels in BioFINDER-1 and WRAP).

	**BioFINDER-1 Cognitively unimpaired**	**BioFINDER-1 Mild cognitive impairment**	**WRAP Cognitively unimpaired**
Plasma biomarkers	uncorrected p-value		
p-tau231	0.946	0.364	0.951
p-tau217	$3.1x10^{-14}$	0.0002	$9.0x10^{-9}$
p-tau181	0.033	0.304	0.021
Aβ42/40	0.806	0.103	0.317
GFAP	0.049	0.030	0.204
NfL	0.184	0.118	0.228

Supplementary Table 2. Uncorrected p-values from Table 2 (Associations between longitudinal plasma biomarkers and longitudinal mini mental state examination (MMSE), modified Preclinical Alzheimer's Cognitive Composite (mPACC), and cortical thickness of the typical AD signature regions in Aβ positive cognitively unimpaired participants in BioFINDER-1 and WRAP).

	**BioFINDER-1**			**WRAP**		
	MMSE	mPACC	Cortical thickness of the typical AD signature regions	MMSE	mPACC	Cortical thickness of the typical AD signature regions
Plasma biomarkers	uncorrected p-value					
p-tau231	0.179	0.456	0.182	0.491	0.356	0.363
p-tau217	0.0001	0.0001	$6.9x10^{-6}$	0.0005	$1.5x10^{-7}$	0.003
p-tau181	0.023	0.158	0.025	0.072	0.159	0.099
Aβ42/40	0.910	0.382	0.505	0.734	0.140	0.204
GFAP	0.029	0.112	0.013	0.754	0.745	0.026
NfL	0.053	0.073	0.191	0.264	0.253	0.011

Supplementary Table 3. Associations of plasma biomarkers with CSF Aβ42/40 (excluding plasma p-tau values below the detection limit) in BioFINDER-1 (cohort 1).

**Plasma biomarkers**	**AUC, 95% CI**	**p-value corrected (uncorrected)**	**AIC**
**Cognitively unimpaired (Aβ+/Aβ-, 95/162)**			
P-tau231	0.883 (0.844-0.923)	NA	226.8
Aβ42/40	0.861 (0.817-0.906)	0.43 (0.43)	237.1
P-tau217	0.800 (0.742-0.858)	0.016 (0.013)	256.2
P-tau181	0.737 (0.675-0.800)	$4.0x10^{-5}$ ($1.6x10^{-5}$)	300.0
GFAP	0.727 (0.663-0.792)	0.0001 ($2.9x10^{-5}$)	324.1
NfL	0.594 (0.524-0.664)	$2.3x10^{-12}$ ($4.5x10^{-13}$)	338.7
**Mild cognitive impairment (Aβ+/Aβ-, 111/49)**			
P-tau231	0.936 (0.895-0.976)	NA	99.2
P-tau217	0.874 (0.814-0.933)	0.10 (0.09)	139.2
P-tau181	0.826 (0.760-0.893)	0.005 (0.004)	153.5
Aβ42/40	0.777 (0.689-0.864)	0.001 (0.0007)	163.1
GFAP	0.723 (0.635-0.811)	$1.3x10^{-5}$ ($4.0x10^{-6}$)	184.5
NfL	0.512 (0.406-0.617)	$2.3x10^{-12}$ ($3.4x10^{-13}$)	200.9

Supplementary Table 4. P-values for biomarkers comparisons in Figure 1 excluding plasma p-tau values below the detection limit).

	**p-tau231**	**p-tau217**	**P-tau181**	**Aβ42/40**	**GFAP**	**NfL**
Q0 (CL <12) [a]	NA	NA	NA	NA	NA	NA
Q1 (CL 12.0-35.9)	3.0e-09 (3.0e-09)	0.402 (0.402)	0.588 (0.588)	0.0447 (0.0447)	0.550 (0.550)	0.906 (0.906)
Q2 (CL 35.9-71.7)	2.3e-10 (1.4e-10)	4.0e-06 (3.0e-06)	0.0002 (0.0001)	1.0e-08 (7.8e-09)	0.0034 (0.0025)	0.721 (0.541)
Q3 (CL 71.7-95.3)	2.3e-10 (1.7e-10)	6.9e-12 (3.5e-12)	4.4e-10 (2.2e-10)	1.3e-09 (3.2e-10)	3.0e-07 (7.4e-08)	0.140 (0.051)
Q4 (CL >95.3)	2.0e-12 (5.1e-13)	1.1e-17 (2.7e-18)	5.5e-12 (1.4e-12)	1.5e-09 (7.3e-10)	8.0e-06 (4.0e-06)	0.140 (0.070)

[a] Reference group

Supplementary Table 5. Associations of Aβ status with longitudinal plasma biomarker levels (excluding plasma p-tau values below the detection limit) in BioFINDER-1 (cohort 2).

**Plasma biomarkers**	**time × Aβ interaction β-estimate**	**time × Aβ interaction p-value corrected (uncorrected)**
**Cognitively unimpaired**		
p-tau231	0.005	0.882 (0.882)
p-tau217	0.260	3.9×10^{-11} (3.3×10^{-12})
p-tau181	0.078	0.008 (0.004)
Aβ42/40	0.007	0.841 (0.806)
GFAP	0.028	0.084 (0.049)
NfL	0.035	0.246 (0.184)
**Mild cognitive impairment**		
p-tau231	-0.073	0.384 (0.304)
p-tau217	0.279	0.001 (0.0005)
p-tau181	0.054	0.395 (0.329)
Aβ42/40	-0.076	0.165 (0.103)
GFAP	0.113	0.059 (0.030)
NfL	0.084	0.177 (0.118)

Supplementary Table 6. Associations between longitudinal plasma biomarkers and longitudinal mini mental state examination (MMSE), modified Preclinical Alzheimer's Cognitive Composite (mPACC) and cortical thickness of the typical AD signature regions in Aβ positive cognitively unimpaired participants (excluding plasma p-tau values below the detection limit) in BioFINDER-1 (cohort 2).

	**MMSE**		**mPACC**		**Word list delayed recall**		**Cortical thickness of the typical AD signature regions**	
	β-estimate	p-value corrected (uncorrected)	β-estimate	p-value corrected (uncorrected)	β-estimate	p-value corrected (uncorrected)	β-estimate	p-value corrected (uncorrected)
Plasma biomarkers								
p-tau231	-0.099	0.227 (0.189)	-0.021	0.528 (0.481)	0.006	0.867 (0.846)	-0.003	0.246 (0.205)
p-tau217	-0.310	0.001 (0.0002)	-0.124	0.001 (0.0002)	0.094	0.028 (0.005)	-0.012	7.6×10^{-5} (1.3×10^{-5})
p-tau181	-0.121	0.182 (0.121)	-0.012	0.528 (0.528)	0.011	0.867 (0.723)	-0.004	0.246 (0.197)
Aβ42/40	-0.010	0.910 (0.910)	0.032	0.528 (0.382)	0.014	0.867 (0.700)	0.002	0.505 (0.505)
GFAP	-0.198	0.087 (0.029)	-0.054	0.223 (0.112)	-0.006	0.867 (0.867)	-0.007	0.040 (0.013)
NfL	-0.194	0.107 (0.053)	-0.067	0.220 (0.073)	0.054	0.474 (0.158)	-0.004	0.246 (0.191)
