# Supplementary material for: Differential roles of Aβ42/40, p-tau231 and p-tau217 for Alzheimer’s trial selection and disease monitoring
Source: Nat Med. 2022 Dec 1;28(12):2555–62. doi: 10.1038/s41591-022-02074-w (PMC9800279; doi:10.1038/s41591-022-02074-w)
Supplement: Supplementary file 2 — Reporting Summary [file 41591_2022_2074_MOESM2_ESM.pdf]

nature portfolio

Corresponding author(s): Kaj Blennow; Oskar Hansson

Last updated by author(s): Sep 23, 2022

Reporting Summary

Nature Portfolio wishes to improve the reproducibility of the work that we publish. This form provides structure for consistency and transparency in reporting. For further information on Nature Portfolio policies, see our Editorial Policies and the Editorial Policy Checklist.

Statistics

For all statistical analyses, confirm that the following items are present in the figure legend, table legend, main text, or Methods section.

n/a	Confirmed	
☐	☒	The exact sample size (n) for each experimental group/condition, given as a discrete number and unit of measurement
☐	☒	A statement on whether measurements were taken from distinct samples or whether the same sample was measured repeatedly
☐	☒	The statistical test(s) used AND whether they are one- or two-sided<br>*Only common tests should be described solely by name; describe more complex techniques in the Methods section.*
☐	☒	A description of all covariates tested
☐	☒	A description of any assumptions or corrections, such as tests of normality and adjustment for multiple comparisons
☐	☒	A full description of the statistical parameters including central tendency (e.g. means) or other basic estimates (e.g. regression coefficient) AND variation (e.g. standard deviation) or associated estimates of uncertainty (e.g. confidence intervals)
☐	☒	For null hypothesis testing, the test statistic (e.g. F, t, r) with confidence intervals, effect sizes, degrees of freedom and P value noted<br>*Give P values as exact values whenever suitable.*
☒	☐	For Bayesian analysis, information on the choice of priors and Markov chain Monte Carlo settings
☒	☐	For hierarchical and complex designs, identification of the appropriate level for tests and full reporting of outcomes
☒	☐	Estimates of effect sizes (e.g. Cohen's d, Pearson's r), indicating how they were calculated

Our web collection on statistics for biologists contains articles on many of the points above.

Software and code

Policy information about availability of computer code

Data collection	No software was used.
Data analysis	SPSS version 28 (IBM, Armonk, NY, US), R studio version 2022.02.0 and R version 4.0.2 (packages pROC and lme4), FreeSurfer image analysis pipeline v6.0 (see http://surfer.nmr.mgh.harvard.edu/

For manuscripts utilizing custom algorithms or software that are central to the research but not yet described in published literature, software must be made available to editors and reviewers. We strongly encourage code deposition in a community repository (e.g. GitHub). See the Nature Portfolio guidelines for submitting code & software for further information.

Data

Policy information about availability of data

All manuscripts must include a data availability statement. This statement should provide the following information, where applicable:

- Accession codes, unique identifiers, or web links for publicly available datasets
- A description of any restrictions on data availability
- For clinical datasets or third party data, please ensure that the statement adheres to our policy

Anonymized aggregated level data will be shared by request from a qualified academic investigator for the sole purpose of replicating procedures and results presented in the article, and as long as data transfer is in agreement with EU legislation on the general data protection regulation and decisions by the Ethical Review Board of Sweden and Region Skåne, which should be regulated in a material transfer agreement.

Human research participants

Policy information about studies involving human research participants and Sex and Gender in Research.

Reporting on sex and gender	We used term "sex" throughout the manuscript. Sex was determined based on self-reporting. Statistical analysis included sex as a covariate; the study included 2 independent cohorts altogether comprised of balanced numbers of man (n=503) and women (n=475); therefore, we believe the findings apply to both sexes.
Population characteristics	Detailed information is given in e-table 1, e-table 2, e-table 4 and eMethods. In short, we present results for analyses from three different cohorts. Cohort 1 and 2 arise both from BioFINDER-1. Cohort 1 and cohort 2 had similar demographic characteristics. Cohort 1 was a cross-sectional analysis and cohort 2+3 included longitudinal plasma samples, imaging, and clinical data. Cohort 1 included 388 cognitively unimpaired participants, 187 mild cognitive impairment (MCI) due to AD. Cohort 2 included a longitudinal analysis of 147 cognitively unimpaired (CU) participants and 95 MCI patients. Cohort 3 included 161 cognitively unimpaired participants. All cohorts include the same, clinical, imaging and plasma biomarker information. Cohort 1 also included CSF biomarker information. In cohort 1, out of 388 CU participants (median (SD) age, 72.2 (5.5) years), 167 were women. In the MCI population, out of 187 participants (median (SD) age, 71.6 (5.4) years), 86 were women. In cohort 2, out of 147 CU participants (median (SD) age, 71.5 (5.1) years), 92 were women. In the MCI population, out of 95 participants (median (SD) age, 70.3 (5.5) years), 36 were women. In cohort 3, out of the 161 participants (median (SD) age, 63.0 (6.2) years), 105 were women.
Recruitment	This project was done as part of the prospective Swedish BioFINDER study. All participants for cohort 1 and cohort 2 were recruited in the prospective and longitudinal BioFINDER-1 study (www.biofinder.se) from 2009 to 2014 in southern Sweden. Recruitment of patients with cognitive impairment or neurological diseases was done at Memory clinics and Neurology clinics. The results for the patients may therefore be biased for a specialist setting. As we already state in the discussion our findings should be validated in a primary care setting. Recruitment of cognitively unimpaired controls was done through advertisements. Cohort 3 is the US Wisconsin Registry for Alzheimer's Prevention (wrap.wisc.edu) who were recruited between 2011 and 2019. Recruitment is described in Johnson et al 2018 DADM and was largely from the community via advertisement.
Ethics oversight	The study was approved by the Regional Ethics Committee in Lund, Sweden. The WRAP data were collected under a University of Wisconsin-Madison Institutional Review Board protocol. All participants in all 3 cohorts gave their informed consent to participate in the study and the data were collected according to the Declaration of Helsinki.

Note that full information on the approval of the study protocol must also be provided in the manuscript.

Field-specific reporting

Please select the one below that is the best fit for your research. If you are not sure, read the appropriate sections before making your selection.

☒ Life sciences ☐ Behavioural & social sciences ☐ Ecological, evolutionary & environmental sciences

For a reference copy of the document with all sections, see nature.com/documents/nr-reporting-summary-flat.pdf

Life sciences study design

All studies must disclose on these points even when the disclosure is negative.

Sample size	The study included two independent prospective cohorts (Swedish BioFINDER-1 study and a subset of the Wisconsin Registry for Alzheimer's Prevention (WRAP) with availability of the requisite data. The BioFINDER-1 was analyzed as a cross-sectional cohort (n=575) and a longitudinal cohort (n=242) with up to 6-years of clinical, imaging, and biochemical data. The WRAP cohort was analyzed longitudinal cohort (n=161) with up to 6-years of clinical, imaging, and biochemical data. Both cohorts were convenience cohorts and all available plasma samples were analyzed in this study. There is no indication that we were insufficiently powered for these analyses.
Data exclusions	The data were limited to the subsets of the source cohorts with available cognitive and biomarker data including plasma assay results. No analyzed samples were excluded from the main analysis but outliers were omitted from figures which are detailed in each legend. Samples <LLOQ were included in the analysis but a sensitivity analysis excluding <LLOQ samples was performed in order to assure that the significant results were not driven by the cases with very low plasma p-tau values. Results of the sensitivity analysis were very similar with the main results and are described in Supplementary Results.
Replication	To verify the findings in the longitudinal BioFINDER-1 cohort we included an independent longitudinal observational cohort from the Wisconsin Registry for Alzheimer's Prevention (WRAP). Even though WRAP participants were was younger, all cognitively unimpaired, and in general had less frequency of co-morbidities, we reproduced the results in both cohorts (all attempts at replication were successful).
Randomization	In these 2 cohort studies (observational studies) no allocation into experimental groups were performed, therefore randomization is not relevant to this study. Statistical analyses were controlled for potential confounding effect of age and sex.
Blinding	All plasma, CSF and PET analyses were performed by individuals who were blinded to the clinical data. Authors who performed the data preprocessing were blinded to demographic and clinical characteristics of individuals.

Reporting for specific materials, systems and methods

We require information from authors about some types of materials, experimental systems and methods used in many studies. Here, indicate whether each material, system or method listed is relevant to your study. If you are not sure if a list item applies to your research, read the appropriate section before selecting a response.

Materials & experimental systems

n/a	Involved in the study
☐	☒ Antibodies
☒	☐ Eukaryotic cell lines
☒	☐ Palaeontology and archaeology
☒	☐ Animals and other organisms
☐	☒ Clinical data
☒	☐ Dual use research of concern

Methods

n/a	Involved in the study
☒	☐ ChIP-seq
☒	☐ Flow cytometry
☒	☐ MRI-based neuroimaging

Antibodies

Antibodies used

Phosphorylated tau 181 (p-tau181): In BioFINDER-1, the p-tau181 assay was performed using phospho specific biotinylated capture antibody (IBA406, developed by Lilly Research Laboratories) and SULFO-TAG- conjugated anti-tau detection antibody (4G10E2, developed by Lilly Research Laboratories). In WRAP, p-tau181 was quantified by the commercial assay from Quanterix.

Phosphorylated tau 217 (p-tau217): In both BioFINDER-1 and WRAP, p-tau217 was quantified in the same manner. Biotinylated-IBA493 (developed by Lilly Research Laboratories) was used as a capture antibody and SULFO-TAG-4G10E2 as the detector.

Phosphorylated tau 231 (p-tau231): In both BioFINDER-1 and WRAP, p-tau231 was quantified in the same manner. For the novel plasma p-tau231 Simoa assay, monoclonal mouse antibodies were generated using a synthetic peptide (K224KVAVVR(pT)PPKSPSSAK240C) as a KLH-coupled antigen, numbered according to full-length tau-441 phosphorylated on threonine 231. Candidate hybridomas were selected on brain extracts of AD and control brain tissue. The final cloned and purified monoclonal antibody, ADx253, was characterized on synthetic peptides spanning amino acids threonine 217 till serine 241 of full-length tau for its affinity, its phospho-specificity using both phosphorylated and non-phosphorylated peptides and its preferred selectivity in which position 232 was replaced by a Pip, to simulate cis-selectivity of ADx253. A biotin-conjugated N- terminal anti-tau mouse monoclonal antibody was used for detection.

Validation

Immunoassay for detection of P-tau181 in human plasma has been previously described by Mielke et al. (Alzheimers Dement 14, 989-997, 2018). Fit for purpose assay validation has been performed by Eli Lilly according to Andreasson et al. (Frontiers in Neurology 2015). P-tau217 immunoassay was been fully described by Palmqvist et al. (JAMA. 2020;324(8):772-781) and p- tau231 by Ashton et al. (Acta Neuropathol. 2021 May;141(5):709-724). Immunoprecipitation and liquid chromatography-mass spectrometry assay for amyloid has been fully described by Schindler et al. (Neurology. 2019 Oct 22;93(17):e1647-e1659).
Commercial assays for amyloid, GFAP and Nfl by Quanterix are propriety but have been widely reported in academic publications (e.g., Thijssen et al., DOI:10.1002/dad2.12285). The Elecsys prototype is propriety but is detailed in a future publication (Palmqvist et al., Alzheimer's & Dementia).

Clinical data

Policy information about clinical studies
All manuscripts should comply with the ICMJE guidelines for publication of clinical research and a completed CONSORT checklist must be included with all submissions.

Clinical trial registration

BioFINDER-1: NCT01208675

Study protocol

BioFINDER-1: https://clinicaltrials.gov/ct2/show/NCT01208675
WRAP: https://wrap.wisc.edu

Data collection

Data were collected between 2009 and 2022. Participating cohorts included BioFINDER-1 (a mix of population-based and memory clinic-based studies in Lund and Malmo, Sweden) and WRAP (a longitudinal observational cohort study enriched with persons with a parental history of probable Alzheimer's disease.

Outcomes

The predefined primary outcome measures are longitudinal changes in plasma biomarker concentrations and their longitudinal changes on MMSE, mPACC and longitudinal brain atrophy. As predefined secondary outcomes we assessed brain β-amyloid positivity and longitudinal changes on delayed recall memory test.
